# Prevalence and antimicrobial resistance profiles of mastitis causing bacteria isolated from dairy goats in Mukurweini Sub‐County, Nyeri County, Kenya

**DOI:** 10.1002/vms3.1420

**Published:** 2024-03-28

**Authors:** Sarah Kabui, Josephine Kimani, Caroline Ngugi, John Kagira

**Affiliations:** ^1^ Department of Biochemistry Jomo Kenyatta University of Agriculture and Technology Nairobi Kenya; ^2^ Department of Medical Microbiology Jomo Kenyatta University of Agriculture and Technology Nairobi Kenya; ^3^ Department of Animal Sciences Jomo Kenyatta University of Agriculture and Technology Nairobi Kenya

**Keywords:** antimicrobial resistance, coagulase‐negative staphylococcus, mastitis, Mukurweini, risk factors

## Abstract

**Background:**

Ruminant mastitis continues to be a cause of economic losses in the dairy industry and remains a major public health hazard globally.

**Objectives:**

This cross‐sectional study was carried out in Mukurweini Sub‐County of Nyeri County, Kenya, to investigate the prevalence of bacteria causing mastitis, risk factors associated with goat mastitis and the antibiotic resistance profiles of bacteria isolated from the goat milk.

**Methods:**

Farm level data on risk factors for mastitis was obtained from 56 farmers using a semi structured questionnaire. A total of 189 goat milk samples were collected. The goat's udder was observed for signs of clinical mastitis and the California Mastitis Test (CMT) used to test the milk for sub‐clinical mastitis. All samples were then cultured for morphological identification of bacteria and strain typing by Matrix Assisted Laser Desorption/Ionization (MALDI)‐Time of Flight (ToF) technique. Antimicrobial susceptibility of the isolated *Staphylococcus aureus*, coagulase‐negative *Staphylococcus* (CoNS), *Escherichia coli*, *Klebsiella oxytoca*, *Pseudomonas* spp., *Enterobacter* spp., *Proteus vulgaris* and *Escherichia vulneris* to eight commonly used antibiotics was done by the disc diffusion method and validated by determining the presence of antibiotic resistance genes (*mecA* and *blaTEM*) using polymerase chain reaction method.

**Results:**

The prevalence of clinical mastitis was 1.1% (2/189) while that of sub‐clinical mastitis was 84.7% (160/189). Higher (*p* < 0.05) prevalence of mastitis was observed in goats whose houses were cleaned fortnightly and in cases where farmers used same towel to dry different does’ udders during the milking process. Thirteen different bacterial species were isolated from the milk samples and identified by MALDI‐ToF, and these included *S. aureus* (22.0%), CoNS (20.3%), *E. coli* (18.1%), *Pseudomonas* spp. (14.3%), *Enterobacter* spp. (10.4%), *K. oxytoca* (6.0%), *E. vulneris* (1.7%), *P. vulgaris* (1.7%), *Raoutella ornithinolytica* (1.7%), *Stenotrophomonas maltophilia* (1.1%), *Pantoea agglomerans* (1.1%), *Serratia marcescens* (1.1%) and *Cedeceas* spp. (0.6%). One hundred pathogenic bacterial isolates were randomly selected and tested for antibiotic sensitivity to eight antibiotics out of which *S. aureus* were 97.5% resistant to Oxacillin and 100% sensitive to Ciprofloxacin. The CoNSs were 100% resistant to Oxacillin and 100% sensitive to Ciprofloxacin. *E. coli* were 93.9% resistant to Oxacillin, 69.7% sensitive to Ciprofloxacin and 87.9% sensitive to both Amoxicillin/Clavulanic acid and Meropenem. The antimicrobial resistant genes detected in *S. aureus* and *E. coli* were *mecA* [66.7%, 0%], and *bla_TEM_* [20% and 78.3%], respectively.

**Conclusion:**

In conclusion, the study showed that most of the does were affected by subclinical mastitis with the main causative bacteria being *Staphylococci* spp. and coliforms. Farmers need to be trained on improved control of mastitis by adoption of good milking practices and use of CMT kit for early detection of mastitis. Occurrence of multidrug resistance by key mastitis causing pathogens was shown to be prevalent and therefore there is need for development of intervention strategies.

## Graphical abstract

The study found out that 1.1% had clinical mastitis while 84.7% had subclinical mastitis. *Staphylococcus* spp. and coliforms were the main causative bacteria and a substantial proportion being resistant to Oxacillin.

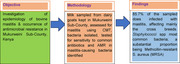

## 1 INTRODUCTION

In Kenya, the dairy goat population is about 200,000, of which 80% are reared by small‐scale farmers in the Mount Kenya region Counties, including Meru, Embu, Nyeri, Kirinyaga and Murang'a (Mbindyo et al., 2018). Mukurweini Sub‐County in Nyeri County has a higher population of dairy goats owing to the combined effort between government and Non‐Governmental Organizations to improve food and nutritional security in the region through dairy farming (Mburu et al., 2014). The social‐economic studies done by Kinyanjui et al. (2008) showed that 57% of the dairy goats’ milk produced was consumed in households, whereas the surplus was sold to specific individuals mainly suffering from ailments, such as diabetes, AIDS and those sensitive to cow milk (Mburu et al., 2014). The same study further noted that in Kenya, there is a high potential of dairy goat farming where each goat can yield up to 2.96 litres of milk daily. However, goat farming is affected by a number of challenges, the main one being infectious diseases such as mastitis (Kagucia et al., 2020; Mahlangu et al., 2018).

Mastitis is the most common disease that affects dairy goats (Gazzola et al., 2021), leading to heavy economic losses associated with poor milk quality, reduced milk yield, high cost of treatment, discarded milk while the animal is on treatment, early culling and sometimes death of the animal (Zigo et al., 2022). Globally, losses due to dairy goat sub‐clinical mastitis have not been well document, but figures range from 65 to 80 Euros (Sánchez etal., 1997; Oosterhuis, 2010, https://aboutsmallruminants.com/analytics‐costs‐mastitis‐sheep‐goats/). The costs are mainly due to production, reproduction losses and treatment costs.

The disease results in the inflammation of the mammary glands as a result of infection by pathogenic agents such as bacteria, fungi or viruses and spreads mainly through unhygienic conditions in dairy farms (Zigo et al., 2022). Contagious mastitis is caused by bacteria residing on the skin of the teat and inside the udder such as *Staphylococcus aureus* and *Streptococcus agalactiae* and is transmissible from one goat to another. Environmental mastitis is caused by environmental pathogens such as *Escherichia coli*, *Streptococcus uberis* and *Klebsiella s*pp., usually found on bedding, feed, manure and soil. Mastitis is, therefore, the outcome of interplay between three major factors: infectious agents, host resistance and environmental factors (Verma et al., 2018). Early detection and effective treatment of mastitis are of great importance to curb losses incurred by farmers while providing nutritional security.

Mastitis is treated by antibiotics, mainly penicillins (Younan, 2002). Although coliform mastitis may sometimes be treated using cephalosporins and fluoroquinolones (Aqib et al., 2019), their use should strictly be based on bacteriological diagnosis (Suojala et al., 2013). Early treatment with effective antibiotics significantly limits the severity of mastitis, economic loss and development of antimicrobial resistance (AMR) (Verma et al., 2018). The overuse and misuse of veterinary antibiotics by farmers has been associated with infection resurgence due to development of AMR in mastitis‐causing pathogens (Aqib et al., 2019). Cloxacillin, for example, an analog of Methicillin/oxacillin, which is used in dry cow therapy, has been implicated in development of methicillin‐resistant *S. aureus* (MRSA) isolates (Saini et al., 2012). The latter has been isolated from raw milk samples and is a leading cause of dairy mastitis. In Kenya, antibiotics are used routinely to treat mastitis in goats (Mahlangu et al., 2018). Worldwide, there has been reduced efficacy of drugs used to treat infections caused by MRSA due to its increased resistance to glycopeptides (Tarai et al., 2013). Further, extended‐spectrum beta‐lactamases (ESBL) antibiotic‐resistant *E. coli* and *Klebsiella* species have been isolated from dairy milk (Badri et al., 2017).

Livestock bacteria can be reservoirs of antibiotic resistance genes such as those associated with ESBL in *Enterobacteriaceae*, which could be transferred to human beings through multiple routes (Ljungquist et al., 2016). In addition to expanded‐spectrum cephalosporins (ceftriaxone, ceftiofur, cefotaxime and ceftazidime), ESBL producers often carry determinants that confer resistance to fluoroquinolones, aminoglycosides and trimethoprim – sulfamethoxazole combination (Saini et al., 2012).

AMR is a public health threat and can potentially cause mortalities approximated to 1 million per annum by 2050 if new effective antimicrobials are not developed (O'Neill, 2016). The mortality rates of infections associated with multidrug‐resistance microorganisms have consistently increased over the last two decades across different populations (Ali et al., 2021). Over time the bacteria that cause mastitis have developed resistance to the antibiotics administered. Knowledge on prevalence of mastitis in dairy goats, microbial diversity, risk factors associated with mastitis development and AMR patterns would greatly improve prevention and guide treatment of the disease.

From the above, it is clear there is an urgent need for extensive research on the status of mastitis and mastitis‐causing pathogens in Kenya so as to improve the existing control measures and to guide treatment. The current study investigated the prevalence and aetiology of clinical and subclinical mastitis, AMR profiles and associated risk factors in dairy goats kept by small‐scale farmers in Mukurweini Sub‐County, Nyeri County, Kenya.

## 2 MATERIALS AND METHODS

### 2.1 Description of the study area

This study was conducted in Mukurweini Sub‐County, Nyeri County, Kenya. The study was conducted in Rugi, Central, West and Gikondi wards.

### 2.2 Study design and sample size calculation

A cross‐sectional study was carried out between August 2021 and April 2022. The sample size was calculated using the formula by Dohoo et al. (2003): *n* = (*Z* ^2^ *αpq*)/*L* ^2^, where *n* is the required sample size, *Zα* is the value of *Z* that provides 95% confidence intervals (1.96), *p* is a priori estimate of the prevalence at the time of the study. With an expected prevalence of 28.6% based on a previous study (Ndegwa et al., 2000), a sample size of 314 was obtained. The minimum sample size of 160 was calculated using the adjusted formula for small animal populations (Thrusfield et al., 2018). In the current study, 189 animals were sampled from a total of 56 farms, each owning 1–10 dairy goats. As there was no formal list of dairy goat farmers available in the study area, the snowball technique was used to identify farmers with lactating dairy goats. The initial farmers were identified by the local extension officers.

### 2.3 Questionnaire survey

During the farm visits, data was collected using a semi structured questionnaire administered on each farm through personal interview by the investigators. The information obtained from the respondents included farm bio‐data and farm management practises such as if the does are housed, floor type, use of beddings, cleaning frequency, washing of udder before milking, drying of udder during milking and use of separate drying towel for each goat during milking. The doe factors which were obtained included breed and history of mastitis.

### 2.4 Milk sample sampling and California Mastitis Test (CMT)

Milk samples were collected aseptically from each teat of 189 lactating dairy goats as described by Deka et al. (2020). With the help of a veterinarian, a detailed visual inspection and systemic palpation of the udder and teats of the lactating doe was done and the milk observed for clinical signs of mastitis. For milk collection, the first three streams of milk were discarded and 3 mL of milk were directly put into a CMT paddle and an equal amount of commercial CMT reagent added (ImmuCell RP). The CMT was observed within 20 s and the results read on a score of 0–3. A score of 0 was considered negative, 1 was considered trace while a score of 2 and 3 was considered positive (Contreras et al., 1996). Ten millilitres of CMT‐positive milk were then collected into labelled sterile universal bottles for further laboratory analysis. The samples were placed in cool boxes with ice packs and transported to the microbiology laboratory within 24 h.

### 2.5 Culture and identification of bacteria

The milk samples were enriched with alkaline peptone water at 37°C overnight. A hundred microliters of the enriched milk samples were inoculated onto both sheep blood agar (Himedia Laboratories) and McConkey agar (Oxoid), respectively. The plates were incubated at 37°C for 24–48 h. Plates which had mixed growth were sub‐cultured to obtain pure colonies. Further characterization was done for the beta haemolytic bacteria obtained from sheep blood agar by catalase test (Reiner, 2010) to differentiate *Streptococcus* from *Staphylococcus* spp.

Strain typing was done using Matrix Assisted Laser Desorption/Ionization (MALDI)‐Time of Flight (ToF) technique. Briefly, forty (40) mg/mL alpha‐cyano‐4‐hydroxycinnamic acid (matrix) (Sigma‐Aldrich) was prepared in LC–MS grade solvents; acetonitrile, ethanol and water in the ratio of 3:3:3 in 3% trifluoroacetic acid. The 25% formic acid overlay method was used for spotting. Using a sterile microcentifuge tip, 0.5 µL bacterial colonies was transferred onto the target plate, and each spot was overlaid with 0.5 µL of 25% formic acid (Sigma‐Aldrich). This was followed by application of 0.5 µL of α‐cyano‐4‐hydroxycinnamic acid matrix onto each spot and thoroughly mixing before MALDI‐MS measurements in Shimadzu Axima Confidence (Shimadzu). A spectrum was acquired for each spot and species identification of each spectrum was compared to the ribosomal marker based database, SARAMIS.

### 2.6 Antibiotic susceptibility testing

Antibiotic susceptibility test was performed using the disc diffusion method (Pum, 2019). One hundred isolates were randomly selected, suspended in growth media, and standardized through a turbidity test (0.5 McFarland's standard). The hundred isolates were considered possible causes of mastitis based on literature. A hundred microlitres of the standardized suspension of the test organism was then inoculated on Mueller Hinton Agar (Himedia, Ltd) plates. Eight antibiotics (Himedia, Ltd) commonly used for treatment of mastitis in Kenya were selected for antibiotic susceptibility testing in the study: Cefuroxime (30 µg), Cefotaxime (30 µg), Amoxicillin and Clavulanic acid (10 µg), Oxacillin (10 µg), Azithromycin (15 µg), Meropenem (10 µg), Ciprofloxacin (10 µg) and Nitrofurantoin (300 µg). The discs were placed on the media surface and plates incubated at 37°C for 24 h. The respective zones of inhibition were measured and results interpreted according to the CLSI (2019) table. Results were recorded as resistant, intermediate or susceptible to specific antibiotics.

### 2.7 Molecular detection of antibiotic resistant genes

Biochemical test was done to identify the bacteria and identity confirmed through MALDI‐ToF technique. Methicillin resistant *S. aureus* was used as a positive control. A total of 35 isolates with multiple antibiotic resistance indexes were selected for molecular detection of antibiotic‐resistant genes. Plasmid DNA was extracted from 35 isolates using plasmid DNA extraction kit (Bioline) following the manufacturer's instructions. Amplification was done using specific primers for detection of genes conferring resistance to Oxacillin (*mecA*), Amoxicillin and Clavulanic acid (*bla_TEM_*) (Mehrotra et al., 2000; Monstein et al., 2010). The primer sequences and the expected fragment size of each gene are listed in Table 1. The polymerase chain reaction amplicons were quantified using a Nanodrop spectrophotometer (Jenway, Genova Nano) followed by the confirmation of a successful recovery by resolving 10 µL of product on 1.5% (w/v) agarose gel and visualizing under UV trans‐illuminator (UVITEC Cambridge) against a 100 bp molecular size ladder.

**TABLE 1:** Primer sequences.

Target gene	Primer sequence	Expected amplicon size (bp)	Source
***bla_TEM_***	F_TCGCCGCATACACTATTCTCAGAATGA R_ACGCTCACCGGCTCCAGATTTAT	445	Monstein et al. (2010)
***MecA***	F_TGG TAT GTG GAA GTT AGA TTG G R_GGA TCT GTA CTG GGT TAA TCA G	166	Mehrotra et al. (2000)

### 2.8 Data analysis

Data entry and management were done using Microsoft Excel 2010 (Microsoft), whereas data analysis was done using SPSS v26 (Microsoft). Descriptive statistical data was presented in tables. Chi square was used to evaluate the association between occurrence of risk factors and prevalence of mastitis (*p* < 0.05). A doe was assumed to be infected with mastitis if it had clinical or sub‐clinical mastitis based on CMT results. Logistic regression was used to test individual risk factors and their strength of association in mastitis infection. The odds ratio was used to determine the strengths of association identified in logistic regression.

## 3 RESULTS

### 3.1 Characteristics of farms and goats sampled

A total of 56 farms were sampled from the study area. The average size of the farms was half an acre with the sizes ranging from quarter acre to three acres majority of the goat houses were raised with timber while some were earthen [35%]. Some (54%) farmers practiced zero grazing, whereas others practiced open grazing (20%) and tethering (26%). The goat breeds kept by the farmers were crosses (34.4%), German Alpine (28.6%), Toggenburg (15.9%), Kenya Alpine (12.2%), Saanen (5.3%) and local breeds (3.7%). The average number of goats kept by farmers was 3 with the number of goats ranging from 1 to 10. Most (84%) of the farmers were not aware of occurrence of mastitis in their dairy goats.

### 3.2 Prevalence of clinical and sub‐clinical mastitis

The overall prevalence of mastitis was 85.7% (162/189) with most (160/189, 84.7%) of them having subclinical disease and a few having clinical mastitis (2/189, 1.1%). Clinical mastitis was characterized by presence of swollen and inflamed udder, presence of flakes in the milk and discoloured milk.

### 3.3 Relationship between prevalence of mastitis and origin of goat, breed milking practices (Table 2)

The results of the CMT were used to evaluate the relationship between mastitis prevalence and risk factors. The highest prevalence of mastitis was in lactating does originating from Central Ward. However, there was no significant difference (*p* = 0.520) between prevalences of mastitis in does originating from the different wards. In terms of breeds, the highest prevalence of mastitis was found in crosses (36.4%), followed by German Alpine (30.9%) and the least affected were the local breeds (1.9%). Nonetheless, there was no significant (*p* = 0.28870) difference in the prevalence of mastitis in the different breeds (Table 3). The highest prevalence of mastitis was in lactating does whose houses were cleaned weekly (*p* = 0.001) compared to those cleaned daily. The highest prevalence of mastitis was found in lactating does where farmers did not wash their hands before milking (*p* = 0.01) compared to those who cleaned their hands. The lowest prevalence of mastitis was found in milk from lactating does whose udder were dried with individual towel (*p* = 0.04) compared to those from farms where towels were shared between different goats (Table 2).

**TABLE 2:** Effect of origin of goat, breed and milking practices on prevalence of mastitis in dairy goat in Mukurweini Sub‐County as identified by California Mastitis Test (CMT) (*n* = 189).

Factor	Number positive	Prevalence of mastitis %
**Ward of origin**		(*p* = 0.52)
Rugi	41	21.69
Central	42	22.22
West	40	21.16
Gikondi	39	20.63
**Breed of goat**		(*p* = 0.2887)
Toggenburg	28/30	14.81
Crosses	59/65	31.22
German Alpine	50/54	26.46
Saanen	7/10	3.70
Kenyan Alpine	15/23	7.94
Local	3/7	1.59
**Pen cleaning schedule**		(*p* = 0.001)
Weekly	71	37.57
Fortnightly	60	31.75
Daily	12	6.35
Irregular	19	10.05
**Hand washing**		(*p* = 0.01)
Yes	142	75.13
No	20	10.58
**Separate drying towel for each goat**		(*p* = 0.04)
Yes	30	15.87
No	132	69.84
**Floor type**		(*p* = 0.35)
Wooden	123	65.08
Earthen	39	20.63
**Use of beddings**		
Yes	0	0
No	162	85.71
**Washing of udder before milking**		(*p* = 0.42)
Yes	153	80.95
No	9	4.76
**Housing**		
Yes	162	85.71
No	0	0

### 3.4 Prevalence of bacteria in the sampled milk

All the 189 samples were cultured. A total of 162 samples positive for clinical and sub‐clinical mastitis yielded bacteria in culture, whereas the 27 samples that were negative for mastitis did not yield any bacteria. The most prevalent species isolated included *S. aureus* (22.0%), coagulase‐negative *Staphylococcus* (CoNS) (20.3%), *E. coli* (18.1%), *Pseudomonas* spp. (14.3%) and *Enterobacter* spp. (10.4%). Other identified species of bacteria and their prevalences are as summarized in Table 3.

**TABLE 3:** Bacteria isolated from dairy goats (*n* = 182) in Mukurweini Sub‐County, Kenya.

Bacteria species	Number of isolates	Percentage
*Staphylococcus aureus*	40	22.0
Coagulase‐negative *Staphylococcus*	37	20.3
*Escherichia coli*	33	18.1
*Pseudomonas* spp.	26	14.3
*Enterobacter* spp.	19	10.4
*Klebsiella oxytoca*	11	6.0
*Proteus vulgaris*	3	1.7
*Raoutella ornithinolytica*	3	1.7
*Escherichia vulneris*	3	1.7
*Pantoea agglomerans*	2	1.1
*Serratia marcescens*	2	1.1
*Stenotrophomonas maltophilia*	2	1.1
*Cedeceus* spp.	1	0.6
Total	182	100

### 3.5 Antimicrobial susceptibility pattern

Antibiotic sensitivity was under taken for the following bacteria; *E. coli*, *Enterobacter* spp., *Klebsiella oxytoca*, *Escherichia vulneris*, *Pseudomonas*, *Proteus vulgaris*, *S. aureus* and CoNS. The tested bacteria were highly resistant to Oxacillin (93.9%–100%). *S. aureus*, which was the most prevalent species, was highly susceptible to Ciprofloxacin (100%), Nitrofurantoin (87.5%) and Azithromycin (82.5%) and highly resistant (97.5%) to Oxacillin. CoNS was 100% susceptible to Ciprofloxacin, 82.2% susceptible to Azithromycin, 68.5% susceptible to Nitrofurantoin and 100% resistant to Oxacillin.

All (100%) *K. oxytoca* isolates were categorized as having intermediate form of resistance. Susceptibility to Amoxicillin/Clavulanic acid was highest in *E. vulneris* (100%), *Pseudomonas* spp. (100%) and *E. coli* (87.9%) and lowest in *K. oxytoca* (72.7%) and *Enterobacter* spp. (57.9%). Strains susceptible to Meropenem included *P. vulgaris* (100%), *E. vulneris* (100%), *K. oxytoca* (90.9%) and *E. coli* (87.9%). *Pseudomonas* spp. isolates were resistant to Azithromycin (100%), Oxacillin (100%) and Ciprofloxacin (100%) and 100% susceptible to Amoxicillin/Clavulanic acid. A summary of the antibiotic susceptibility profiles is given in Tables 4 and 5.

**TABLE 4:** Antibiotic susceptibility profiles of *Escherichia coli*, *Enterobacter* spp., *Klebsiella oxytoca*, *Escherichia vulneris*, *Pseudomonas* spp. and *Proteus vulgaris* (percentages) isolated from lactating does in Mukurweini Sub‐County (*n* = 95).

	*E. coli* (*n* = 33)			*Enterobacter* spp. (*n* = 19)			*K. oxytoca* (*n* = 11)			*E. vulneris* (*n* = 3)			*Pseudomonas* spp. (*n* = 26)			*Proteus vulgaris* (*n* = 3)		
Antibiotics	*R*	*S*	*I*	*R*	*S*	*I*	*R*	*S*	*I*	*R*	*S*	*I*	*R*	*S*	*I*	*R*	*S*	*I*
AMC	0	87.9	12.1	21.1	57.9	21.1	9.1	72.7	18.2	0	100	0	0	100	0	0	66.7	33.3
CXM	12.1	15.2	72.7	26.3	15.8	57.9	18.2	18.2	63.6	66.7	33.3	0	50	50	0	33.3	0	66.7
CTX	27.3	57.6	15.2	26.3	47.4	26.3	18.2	72.7	9.1	66.7	33.3	0	50	50	0	33.3	66.7	0
Mpm	6.1	87.9	6.1	5.3	15.8	78.9	0	90.9	9.1	0	100	0	0	50	50	0	100	0
FT	3.0	51.5	45.5	26.3	42.1	31.6	18.2	63.6	18.2	66.7	33.3	0	50	50	0	66.7	0	33.3
AZM	30.3	69.7	0	10.5	31.6	57.9	36.4	63.6	0	33.3	66.7	0	100	0	0	66.7	0	33.3
OX	93.9	6.1	0	100	0	0	0	0	100	100	0	0	100	0	0	100	0	0
CIP	12.1	69.7	18.2	15.8	15.8	68.4	0	90.9	9.1	33.3	66.7	0	100	0	0	0	100	0

Abbreviations: AMC, Amoxicillin + Clavulanic acid; AZM, Azithromycin; CIP, Ciprofloxacin; CTX, Cefotaxime; CXM, Cefuroxime; FT, Nitrofurantoin; *I*, intermediate; Mpm, Meropenem; OX, Oxacillin; *R*, resistant; *S*, susceptible.

**TABLE 5:** Antibiotic susceptibility profiles of *Staphylococcus aureus* and coagulase‐negative *Staphylococcus* (CoNS) (in percentages) isolated from lactating does in Mukurweini Sub‐County (*n* = 77).

	*S. aureus* (*n* = 40)			CoNS (*n* = 37)		
Antibiotics	*R*	*S*	*I*	*R*	*S*	*I*
Nitrofurantoin	12.5	87.5	0	32.4	67.6	0
Azithromycin	17.5	82.5	0	18.9	81.1	0
Oxacillin	97.5	2.5	0	100	0	0
Ciprofloxacin	0	100	0	0	100	0

Abbreviations: CoNS, coagulase‐negative *Staphylococcus*; I, intermediate; S, susceptible; R, resistant.

### 3.6 Detection of antimicrobial resistance genes

**FIGURE 1:**
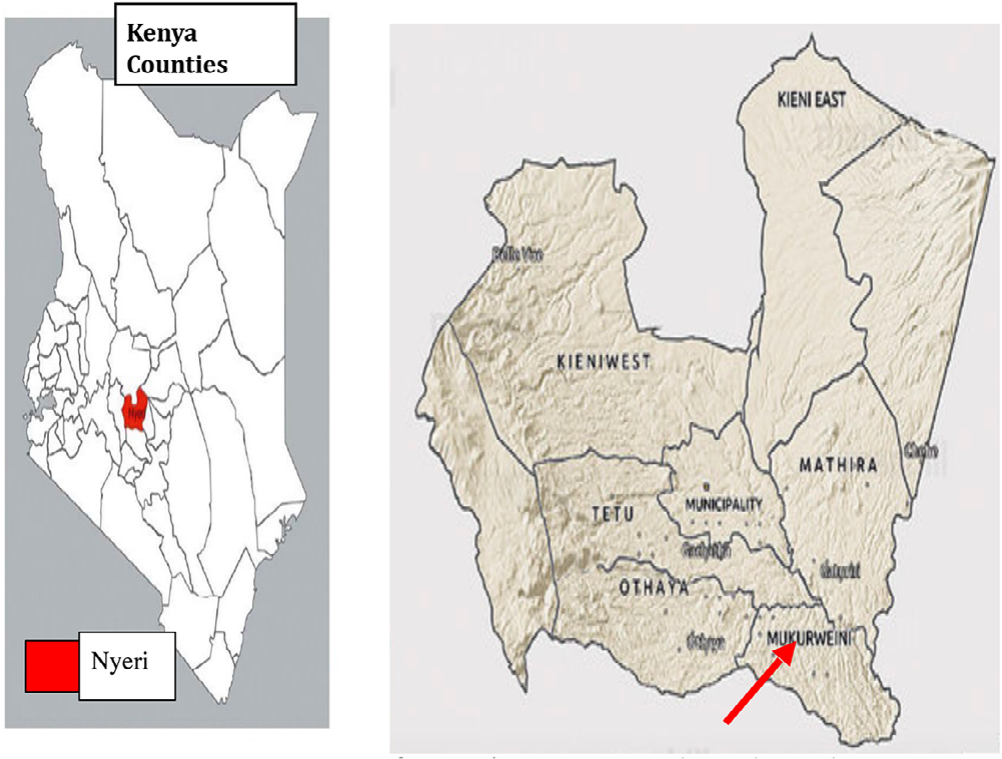
Map of Kenya showing the location of Nyeri County and study Sub‐County. Mukurweini Sub‐County has an area of 171.1 km^2^ with a population of 89,137 people and a density of 498 people per square kilometre. The coordinates for Mukurweini Sub‐County are 0.5609° S, 37.0488° E. The average temperature of Mukurweini is a high of 24°C and a low of 13°C. The average annual rainfall in Mukurweini is 1206 mm and occurs in two seasons. The livestock population of Mukurweini Sub‐County is 168,685 (Kenya National Bureau of Statistics, 2019), comprising of exotic cattle, indigenous cattle, sheep, goats, donkeys, pigs and chicken. The goat population was 10,379 as per the 2019 national census (Kenya National Bureau of Statistics, 2019).

**FIGURE 2:**
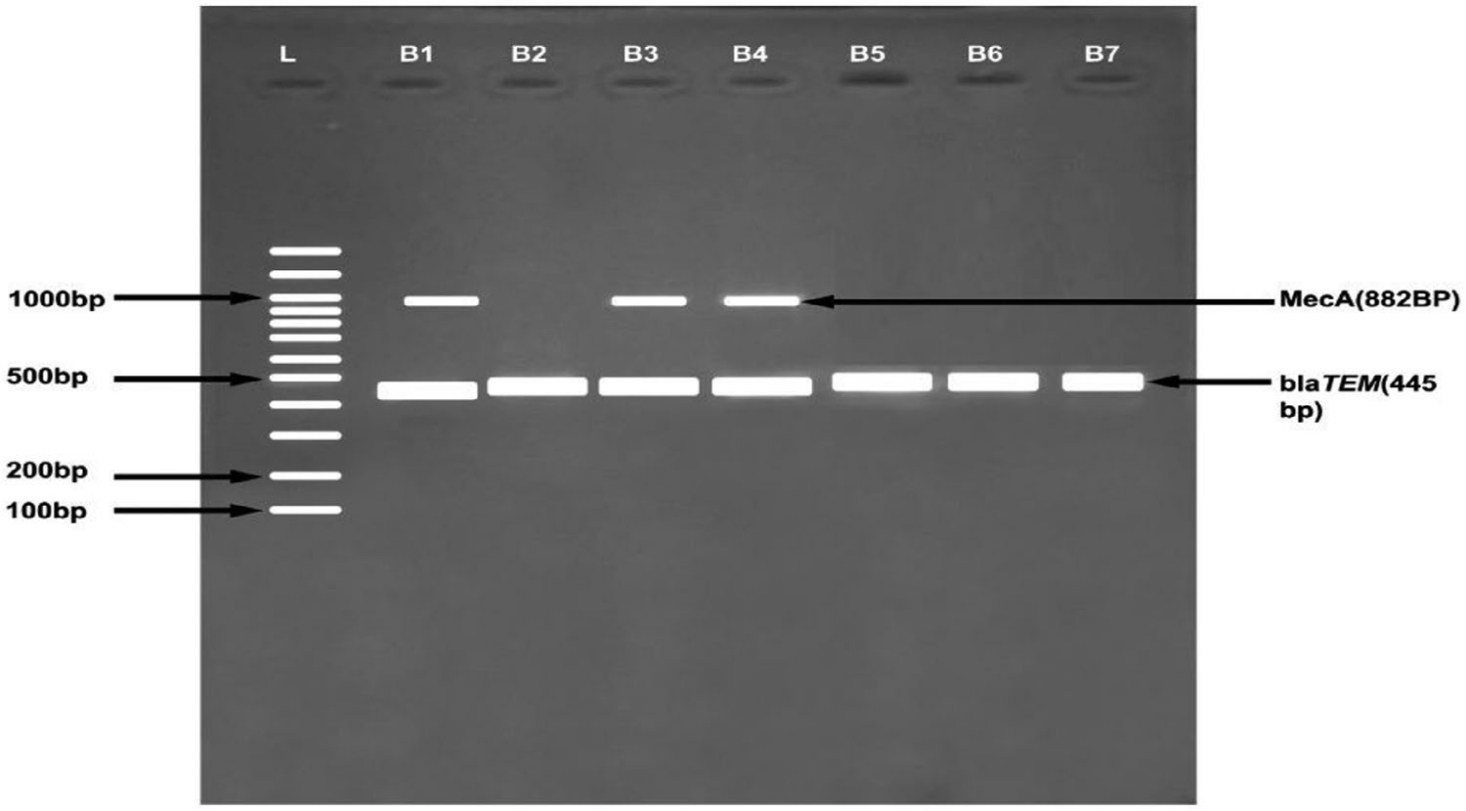
Gel image showing MecA and blaTEM: (B1 and B2) *Escherichia coli*, (B3 and B4) *Staphylococcus aureus*, (B5 and B6) *Enterobacter*, (B7) *Klebsiella oxytoca* and (L) hyperladder 100 bp Bioline.

Bla*_TEM_* antibiotic resistance genes were detected in the plasmid DNA of *E. coli, Enterobacter* spp. and *K. oxytoca* (Figure 1). MecA and Bla*_TEM_* antibiotic resistance genes were detected in *S. aureus* isolates. The bands on the agarose gel gave the expected sizes of 445 and 225 bp, respectively. A total of 18 *E. coli* isolates (78.3 %) showed the presence of *bla_TEM_*, whereas no (0%) isolates had *MecA* gene. *MecA* and bla*_TEM_* genes were detected in 10 and 3 *S. aureus* isolates, respectively, as shown in Figure 2.

## 4 DISCUSSION

The current study sought to characterize mastitis‐causing pathogens and their antibiogram in dairy goats reared by small‐scale farmers from Mukurweini Sub‐County in Nyeri County, Kenya. Our study found the prevalence of sub‐clinical to be higher than clinical mastitis, which is expected because sub‐clinical mastitis cases are not detected by physical examination of milk or udder. The current study agrees with a study in Pakistan (Mirza et al., 2017), which reported a prevalence of mastitis to be 75%–85% over a period of 3 years. The findings are also close to the findings of Jabbar et al. (2020), which reported the overall prevalence of mastitis in Pakistan to be 61.8%. Compared to other Kenyan studies, the prevalence of mastitis in the current study area was higher than that reported by both Ndegwa et al. (2000) (9.8%) and Mahlangu et al. (2018) (50.9%) in dairy goats from Thika East Sub‐County, Kenya. This study revealed that farms that did not use an udder drying towel for each cow had significantly higher prevalence of mastitis than farms that used a drying towel for each cow. This agrees with finding by Mbindyo et al. (2020) from Mekonnen et al. (2017) from Ethiopia who reported that use of the same drying towel for the herd was responsible for the spread of mastitis‐causing pathogens. This can explain the high prevalence of *Staphylococcus* spp. reported in this study. As these organisms are part of the normal flora residing in the udder and the teats, they can spread through the use of same drying towel during milking. In this study, there was significantly higher prevalence of mastitis in does whose houses were cleaned weekly compared with does whose houses were cleaned more frequently. These results are in agreement with those by others (Mahlangu et al., 2018; Mbindyo et al., 2014). The study showed hygiene plays a major role in occurrence of subclinical mastitis. Farms where the hands were not washed before milking showed significantly higher prevalence of mastitis compared with farms where farmers washed hands before milking (Gianneechini et al., 2002). In contrast to other studies (Mahlangu et al., 2018; Mbindyo et al., 2014), the current one found that cross‐breeds had higher prevalence of mastitis compared to others breeds of goats. The causes of variation in the breed differences are not clear and should be studied further.

The current study found CoNS, *S. aureus, E. coli* and *Enterobacter* spp. to be the predominant bacteria causing mastitis in goats. Our findings are consistent with those of Mahlangu et al. (2018) who found CoNS (20.7%) and *S. aureus* (10.7%) as the commonest causative agents of mastitis in dairy goats kept by farmers in Thika Sub‐County, Kenya. Our findings are also consistent with the findings of Saidani et al. (2018) which found CoNS (20.7%), *S. aureus* (10.7%) among the Gram‐positive bacteria, *Enterobacter* spp. (6.5%) and *E. coli* (5.9%) among the Gram‐negative bacteria as major causative agents of mastitis in Indonesia. Altaf et al. (2019) and Jabbar et al. (2020) also found *Staphylococcus* spp. to be the predominant bacteria causing subclinical mastitis in goats in Pakistan. Pisanu et al. (2020) stated that *Staphylococcus* spp. are the most prevalent intramammary pathogens in dairy goats causing subclinical, clinical, acute and gangrenous mastitis. Further, in recent years, it is important to note that CoNS have emerged as a significant threat to food safety since they have been shown to harbour numerous enterotoxins and AMR genes (Gizaw et al., 2020).

Coliforms, including *Klebsiella* spp*., E. coli* and *Enterobacter* spp. were also observed in dairy goats in the current study. These bacteria are normally associated with environmental contamination of the udder and establishment of intramammary infection. The presence of coliforms may be linked with poor hygiene practices in dairy goat farms (Okoko et al., 2020). In our study, the sharing of towels to dry the udders, failure to wash hands before milking and infrequent cleaning of goats’ houses were found to be risk factors for the development of mastitis in the lactating goats. This agrees with the study done by Mbindyo et al. (2020) in Embu and Kajiado Counties in Kenya, which showed that the use of same drying towel for the herd was responsible for spreading mastitis pathogens.

In the current study, the isolated *E. coli* showed high antibiotic resistance to oxacillin but high susceptibility to Amoxicillin/Clavulanic acid, Meropenem, Azithromycin and Ciprofloxacin. Multidrug resistance has been observed in the Gram‐negative bovine mastitis pathogens (Saini et al., 2012). In the current study, multidrug resistance was found in *E. coli* isolates (resistant to Oxacillin, Cefotaxime, Azithromycin, Cefuroxime, Meropenem, Nitrofurantoin and Ciprofloxacin), *Enterobacter* species (resistant to Cefuroxime, Cefotaxime, Amoxicillin/Clavulanic acid, Meropenem, Nitrofurantoin, Azithromycin, Ciprofloxacin and Oxacillin) and *K. oxytoca* isolates (resistant to Cefuroxime, Cefotaxime, Amoxicilli/Clavulanic acid, Nitrofurantoin and Azithromycin). Coliform contaminations rank high among the most types of contamination in the dairy industry. Microorganisms, such as *E. coli*, *Pseudomonas aeruginosa*, *Citrobacter* spp., *Klebsiella* spp. and *Proteus mirabilis*, can multiply in the normal summer temperatures, and hence unpasteurized milk has every chance of containing *E. coli* (Dhanashekar et al, 2012). Amoxicillin/Clavulanic acid was also found to be effective against *Enterobacter* spp., *E. vulneris*, *Pseudomonas* spp. and *P. vulgaris*. This can be attributed to limited use of Amoxicillin/Clavulanic acid in the treatment of sub‐clinical mastitis in the current study area.

Most bacteria isolates from the current study area were resistant to Oxacillin. Most *S. aureus* isolates were resistant to Oxacillin. *S. aureus* is an important pathogen because of a combination of toxin‐mediated virulence, invasiveness and antibiotic resistance (Guimarães et al., 2017). Moreover, the bacterium is a significant cause of human nosocomial infections (Guimarães et al., 2017). On the contrary, *S. aureus* showed high antibiotic susceptibility to Ciprofloxacin, Nitrofurantoin and Azithromycin. The findings on susceptibility agrees with a study by Upadhyay and others that reported high susceptibility of *S. aureus* to Azithromycin (100%) and Ciprofloxacin (76.67%) (Upadhyay & Kumar Kataria, 2009).

The current study focused on detection of AMR genes in dominant species causing mastitis. *E. coli* has the ability to produce ß‐lactamase enzyme and modified penicillin‐binding proteins that hydrolyse or inhibit the binding of ß‐lactam antibiotics (Okoko et al., 2020). In *S. aureus*, the current study reported the presence of *mecA* gene while in *E. coli* bla*_TEM_* antibiotic resistance gene was identified. Similar findings in *E. coli* and *S. aureus* were reported by other authors (Eltaweil et al., 2022; Milk & Gelbı, 2018). These resistant genes are common and have been shown to spread across the ecosystem (man–animal–environment) through horizontal gene transfer (Todorović et al., 2018). *Enterobacter* spp. are common human gut micro flora but have also been isolated from milk (Hogan & Smith, 2012). In our study, *Enterobacter* spp. was found in goat milk, suggesting the possible transmission of the bacteria to goat udders through water, soil or faecal contamination. *Enterobacter* spp. and *E. coli* are highly resistant to Oxacillin. Our study agrees with a study done in India which showed *E. coli* and other mastitis bacteria to be highly resistant to Oxacillin (Fahim et al., 2019).

Some bacteria that were found in the milk samples such *Stenotrophomonas maltophilia* are environmentally acquired opportunistic pathogens which are not known to cause mastitis. Other studies have found this bacteria to carry resistance genes to most classes of antibiotics, including beta‐lactamases, aminoglycoside inactivating enzymes and efflux pumps (Gil‐Gil et al., 2020). Its presence in milk, therefore, is a major public health concern as it is known to cause a number of infections mainly at hospitals (among immunocompromised hosts) as well as in cystic fibrosis patients (Gil‐Gil et al., 2020) and can potentially contribute to transfer of AMR genes from *S. maltophilia* to humans through milk consumption. In recent years, *K. oxytoca* has been reported as a significant opportunistic pathogen causing nosocomial infections in neonates as well as adults (Neog et al., 2021). *Klebsiella* species have been found to harbour many AMR genes, including *blaTEM*, *bla OXA* and *blaCTM‐M*‐*15* (Muraya et al., 2022). The most prevalent ESBL‐producing Enterobacterales in Kenya are *CTX‐M‐15* and *TEM* which is associated with use of third generation Cephalosporins in a previous study (Muraya et al., 2022). In our study, *K. oxytoca* was found to be resistant to Amoxicillin/Clavulanic acid, Cefuroxime and Cefotaxime which agrees with a study done by Neog et al. (2021) in India.

## 5 CONCLUSIONS

The current study found a high prevalence of sub‐clinical mastitis in dairy goats in the study region. The major predisposing factors to goats contracting mastitis were poor hygiene factors including the use of one towel to dry several udders during milking and not cleaning the goat pens frequently. To mitigate emergence and spread of mastitis, farmers should be trained on proper hygiene practices including daily cleaning of goat pens and using one towel per doe to clean the udders during milking. There is also need to train farmers on use of CMT in order to have early detection of mastitis. Majority of the bacteria causing mastitis were resistant to the common antibiotics used to treat mastitis, particularly to Oxacillin. Other drugs were found to have high efficacy and thus could be recommended for treatment of mastitis in the study area, and these include Ciprofloxacin, Meropenem, Nitrofurantoin and Azithromycin. Further studies should be undertaken to determine the causes of the high burden of AMR and whether there could be cross‐transmission of these bacteria to humans.

## AUTHOR CONTRIBUTIONS

*Designing of the study, data collection and analysis, writing the original draft, review and editing of the manuscript*: Sarah Kabui. *Funding acquisition and study supervision, analysis of molecular study data and critical revision of the manuscript for important intellectual content*: Josephine Kimani. *Funding acquisition and study supervision, analysis of the microbiology data and critical revision of the manuscript for important intellectual content*: Caroline Ngugi. *Funding acquisition and study supervision, designing the study, analysis of the mastitis and antibiotic sensitivity data and critical revision of the manuscript for important intellectual content*: John Kagira.

## CONFLICT OF INTEREST STATEMENT

The authors declare that they have no financial or personal relationships that may have inappropriately influenced them in writing this article.

### PEER REVIEW

The peer review history for this article is available at https://www.webofscience.com/api/gateway/wos/peer‐review/10.1002/vms3.1420.

## ETHICS STATEMENT

Ethical approval was obtained from the Ethics Committee of Institute of Primate Research, Kenya. Milk samples were obtained with the help of the farmers who volunteered to participate in the study under the supervision of veterinary officers from the county veterinary office. The protocols used for sampling, bacterial isolation and antibiotic susceptibility testing were performed in agreement with the Animal Diseases act, Section 20, Act of 1984 (Act No. 35 of 1984).

## Data Availability

The data collected and analysed during this study are available from the corresponding author on reasonable request.
